# Nursing Students’ Knowledge, Awareness, and Experiences of Emergency Contraception Pills’ Use

**DOI:** 10.3390/jcm11020418

**Published:** 2022-01-14

**Authors:** Fatima Leon-Larios, Cecilia Ruiz-Ferron, Rocio-Marina Jalon-Neira, Juan-Manuel Praena-Fernández

**Affiliations:** 1 Nursing Department, Faculty of Nursing, Physiotherapy and Podiatry, University of Seville, 41009 Seville, Spain; fatimaleon@us.es (F.L.-L.); jpraena_2@ugr.es (J.-M.P.-F.); 2 University Hospital Virgen del Rocío, 41013 Seville, Spain; 3 Aljarafe District, Health Andalusian Service, 41917 Seville, Spain; marina_jalon@hotmail.com; 4 Unit Biostatistic, Department of Statistics, Faculty of Medicine, University of Granada, 18016 Granada, Spain

**Keywords:** emergency contraception pills, morning-after pill, levonorgestrel, ulipristal acetate, sexual behavior, students, contraceptive knowledge, contraceptive attitude, contraceptive experience

## Abstract

The emergency contraception pill (ECP) is a non-prescribed medication in Spain. However, there is not enough evidence of its use among young people to define sex education contents. The aims of this research were to describe the experiences of the use of the ECP in university students and analyze their knowledge, attitude, and awareness regarding the ECP. The cross-sectional, analytic study was conducted with nursing degree students at the University of Seville. A total of 478 students answered the questionnaire. All of the students (100%) had heard about the ECP and had a positive attitude towards this contraceptive. A total of 25.7% had used the ECP, mainly because a condom had failed or because they did not use any contraceptive at all. Deficiencies in knowledge are related with the ECPs’ mechanism of action, efficacy after repeated use, and the type of ECP available. Female students who used no method at all or withdrawal, and who were over 20 years old, used ECP to a greater extent (*p* < 0.005). Further education initiatives focused on the use of the ECP, its efficacy, and typology are needed, particularly among future health professionals who will later educate other young people.

## 1. Introduction

Emergency contraception (EC) is used to prevent pregnancy if the chosen contraceptive has failed or if no contraception was used. Under no circumstances must it be considered a contraception method to be used on a regular basis. The use of an emergency contraception pill (ECP) is recommended as soon as possible whenever sexual intercourse with a chance of pregnancy has occurred, preferably within the first 12 h postcoitum, the first 72 h if levonorgestrel (LNG) is administered, or within the first 5 days when using ulipristal acetate (UPA). The mechanism of action of EC is avoiding pregnancy, inhibiting or delaying ovulation, but not preventing implantation of a fertilized egg. This drug will neither cause an abortion nor embryo damage [1]. However, it must be taken into account that this drug does not offer any protection against sexually transmitted infections (STI) [2,3].

The distribution of the ECP in Spain has changed in the last years; it can be administered in pharmacies without prescription, since 2009 for LNG and since 2015 for UPA [4]. This facilitates access to young people [3], though the vulnerable population may face difficulties in this access as the dispensation cost of this drug must be borne by the user [5]. The fact that access and purchase are easy means that young people must have information on its usage, effects, and management [6]. Despite its common use, around 30–50% of young people under the age of 30 have used the ECP at some point in their lives, knowledge about this drug is scarce [7,8,9,10]. The use pattern is related to its availability and accessibility in combination with individual knowledge and awareness of ECP [9,11].

Sex education training nowadays has proved to be poor [12,13,14], and in many cases it does not include information on the ECP. Sexual education has been proven to impact sexual behavior, the latter becoming healthier [15,16]. Even in the setting of university stages, sex education in contraception and sexual health is needed, particularly for health professionals who can include sex education in their teaching activities [17,18]. Notwithstanding this, it has been observed that in studies related to Health Science, students develop healthier behaviors and acquire more knowledge in this field than those students pursuing their degrees in other fields [13].

The objectives of this study were basically three: (1) describe the experiences of ECP users at university stages; (2) analyze their knowledge on ECP; (3) define both attitudes and awareness around ECP with the aim of designing educational programs at university level.

## 2. Materials and Methods

### 2.1. Study Design

A cross-sectional, analytic, descriptive study was carried out. Participants were male and female students of the nursing degree during the academic years 2019–20 and 2020–21 at Universidad de Sevilla. Male students were asked about their experience with their partners regarding ECP use. The questionnaire was performed prior to the seminar on family planning programmed for the subject Nursing in Reproductive Health, which includes content related to ECP use. The aim was to identify any lack of knowledge, level of awareness, plus attitudes and experiences regarding the ECP.

### 2.2. Measures

The questionnaire administered online was created ad hoc based on the existing literature [8,19,20,21,22,23] by the first two authors and agreed by the four authors. Socio-demographic variables of sex, age, and sexual orientation were included. Students were asked about their sexual behavior, ECP use and reasons to use it, time gap after sexual intercourse and use, ECP use recommendations to others, symptomatology associated with ECP use, and if they felt embarrassed when they purchased it at the pharmacy.

Moreover, students were asked about their knowledge of the ECP (10 questions posed in affirmative and negative forms that could be answered with true, false, do not know/no answer) and its mechanism of action and effects. Each correct answer scored 1 point, and each wrong answer or no answer added 0 points, each participant obtained a mark out of a total score of 10 points.

### 2.3. Data Collection

Data regarding the period October 2019–October 2020 were collected through an online questionnaire via Google Drive. This questionnaire was pilot tested by 10 students in order to identify potential weaknesses. Once the review process ended, the final version was launched. Students were provided with a link to a questionnaire form to be completed from their mobile devices (phone or laptop). 

### 2.4. Ethical Aspects

Anonymity and confidentiality when answering the questionnaire was granted at all times. Prior to the completion of the questionnaire, students were informed about the objectives of the study and they also were asked for their consent to participate with their data in the study. All the information was provided by the students. Ethical review and approval were waived due to the research being an anonymous web-based study, thus it did not require approval.

### 2.5. Data Analysis

An exploratory data analysis was performed in order to identify any possible outliers. This analysis took into account the whole population studied, as well as the different subgroups created. An exploratory graph analysis of the different studied variables according to their numeric or non-numeric character was conducted. Frequency distribution and percentages were used for the descriptive analysis of qualitative (non-numeric) variables. For quantitative (numeric) variables, centrality and dispersion statistics were calculated.

The relation between two qualitative variables was analyzed through Pearson’s chi-square test, Yates’s chi-square test, linear-by-linear association test, and Fisher’s exact test, according to mandatory criteria. Significant results of these tests were completed with a 95% confidence interval (CI) for percentage differences.

With respect to numeric variables between two groups, the Student’s *t*-test was applied to compare the means, the requirements of normality and randomness were validated (Kolmogorov–Smirnov test or Shapiro–Wilks test according to sample size) and the F-test was used for equality of variances (Levene’s test). When the latter did not work, the Student’s T-test was performed for independent samples with Welch’s correction. When normality requirements were not met, the Mann–Whitney U test was applied.

For the comparison of numeric variables between more than two groups, and once the requirements of independence, normality, and homoscedasticity were validated, the ANOVA test (Analysis of variance) was applied. When requirements were not met, the Kruskal–Wallis test was applied. In order to determine the risk factors associated with EC use, a logistic regression model was applied obtaining both the crude and adjusted odds ratio at a 95% confidence interval.

In all hypothesis contrasts, the significance level was set at 0.05. Data analysis was processed by IBM SPSS 26 (IBM Corp., Armonk, NY, USA).

## 3. Results

A total of 478 students took part in the study during the academic years 2019/20 and 2020/21. All participants accepted to answer the questionnaire in the first 10–15 min of the workshop where they were going to receive information about contraception as part of the curricula of the nursing degree, so the rate of response was 100%. No student refused to participate.

The majority of participants were under the age of 25, with an average age of 20.87 (SD 4.52). Participants’ socio-demographic variables are indicated in Table 1, as well as sexual behaviors, and sex-disaggregated contraceptive use. 

The average age for first sexual encounters was 16.53 (1.46), differentiating males (16.99 ± 1.57 years) and females (16.41 ± 1.46) years, *p* < 0.001, (IC 0.225–0.924). Male students declared themselves more sexually active than their female peers, 83.9% of participants allegedly having any kind of sexual intercourse. In opposition to national statistics, in this sample, male students proved to have their first sexual encounter later than girls. 

**Figure 1:**
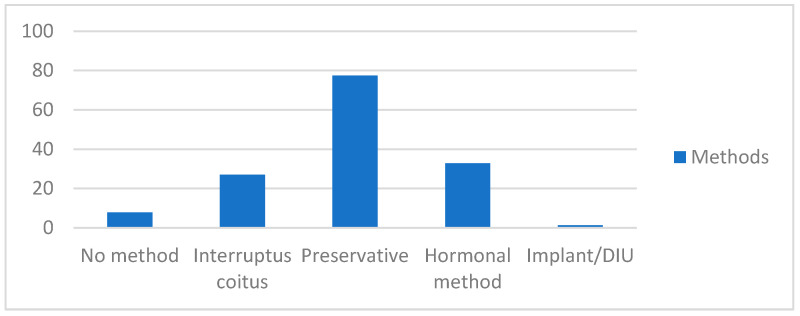
Contraceptive methods used at some point of the sex life (%).

Participants were asked for the contraceptive methods used throughout their sex life. The most common contraceptive method is the condom (77.4% (370)), followed by hormonal methods (pill, patch, and ring) (32.8% (157)). A high percentage of young people still use coitus interruptus as a method to avoid pregnancy (27% (129)), and almost 7.9% (38) do not use any method during their sexual relations as shown in Figure 1.

**Figure 2:**
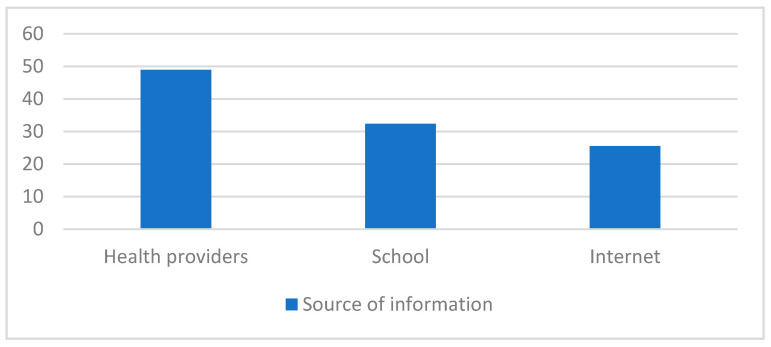
Previous education on contraceptive methods.

In total, 7 out of 10 students declared they had been provided with some type of information on contraception methods, including the emergency contraceptive pill. The major information sources of those who acknowledged having received previous training were via health professionals (48.96% (165)), the Internet (32.34% (109)), and at school (25.51% (86)), as shown in Figure 2.

### 3.1. ECP Use and Attitudes

In all, 100% of participants showed awareness about ECP. A total of 25.7% (123) had used the ECP: 69.1% (85) of these just once, 19.5% (24) two times, 10.6% (13) three times, and 0.8% (1) up to five times. There were some significant differences (*p* < 0.001) between female (34.7%) and male students (15.5%).

The main reason indicated for using the ECP was due to any issue regarding the condom (it broke or came off) (3.41%, 78 participants), 37.77% (44) did not use any method consistently during sexual intercourse or tried ineffectually a withdrawal/coitus interruptus. After using the ECP, 33.3% (41) changed to another contraceptive method. A total of 33.3% (41) of ECP users had after effects after the use of the ECP, the most common being nausea/vomiting (34.78% (16)), irregular bleeding (28.26% (13)), general unrest (23.91% (11)), and headache (13.04 (6)). Despite female students suffering from these symptoms, a total of 58.5% (24) would recommend its use to other women if they needed it. In general terms, 80.5% (99) of participants using the ECP did not recall which type of pill it was, and 77.2% (95) used it within the first 12 h, regardless the student’s sex or age (*p* = 0.72).

Female participants who used the ECP had their first sexual intercourse at a younger age (15.76 (1.31)) than those who did not (16.87 (1.40)), which was statistically significant (*p* < 0.001). In total, 82.9% (102) had a steady partner when they used the ECP, and 62.6% (77) felt embarrassed when they had to purchase it at the pharmacy. Approximately two-thirds (66.7% (82)) of participants who used it would recommend it use to others, with similar results among female and male students. There is no relation between having received previous information on the ECP and its use: 27.1% did not receive any information and 32% did (*p* = 0.392).

More than half of the participants (58.6% (280)) considered that the ECP should be free, with no significant differences in sex or opinion among people who had used them and those who did not (*p* = 0.12). Only three students (2.4%) in the sample had undergone a voluntary termination of pregnancy, and all of them had used the ECP at some point in their lives. One of these students had used the ECP once; the other two, more than once. The age of their first sexual encounter was below the sample mean.

### 3.2. Knowledge about ECP

Questions about the ECP are indicated in Table 2, the students’ answers segregated by sex. Percentage of success is indicated. It is observed that 4 out of 10 men thought that the ECP mechanism of action is abortion, and 5 out of 10 men considered the ECP a contraceptive method. Almost half of the female and male students considered that the ECP may harm the embryo if the woman is pregnant.

The use of ulipristal acetate as an oral emergency contraceptive within the first 5 days after the risk of pregnancy was known by 5 out of 10 students. There was an erroneous belief that ECPs reduce their effect with repeated use.

Most participants were aware of the fact that the use of ECPs does not protect from STIs, and knew that neither a medical prescription nor a pregnancy test are needed for its use. Between 7–8 of 10 students were aware that the time after sexual intercourse is crucial for the efficacy of the ECP.

There is no relation between the age when the first sexual encounter takes place and the score obtained in the test (*p* = 0.069). However, the students ranking higher in the questionnaire were those who had received previous information in the subject, 6.42 vs. 5.81 over 10 scores (*p* < 0.001). Likewise, female students who had received any kind of sex education on this aspect obtained higher scores (6.51 vs. 5.86, *p* < 0.001), while male students’ answers show no statistically significant differences (6.06 vs. 5.57, *p* = 0.209).

Students who had used the ECP obtained higher scores in the knowledge test (6.61 ± 1.56 vs. 6.13 ± 1.68), *p* = 0.007). The level of knowledge regarding each item does not condition the recommendation of the use of the ECP to females that may need it (*p* > 0.05).

Table 3 illustrates the factors independently associated to ECP use are age (20 years old), sex (woman), contraceptive method used, and knowledge about ECP. These factors jointly maintain the risk of significant use.

## 4. Discussion

The aim of this study was to explore the knowledge, attitudes, awareness, and experiences of university students of the nursing degree at the Universidad de Sevilla regarding ECP, in order to define the educational action needed as future health agents educating young people in this matter.

An increased use of the ECP was expected with the arrival of deregulated access to ECPs in pharmacies. However, it has been observed that there had only been a slight increase that had disappeared over the years [24] or the use had remained stable [8]. Results of this study revealed that the totality of the students had heard about EC, as also observed in other studies [25]. The prevalence of drug use in this sample (25.7%) is similar to other published studies with Spanish cohorts [12,17], and those from other countries [10,26]; however, this prevalence is lower than that observed in other studies [19,27]. Almost all participants were knowledgeable about the fact that the ECP required no medical prescription for its purchase and use; other studies with university students showing similar results [26,28].

Access to ECPs is more difficult when people lack the knowledge or the socioeconomic resources [5,29]. All participants in the sample were university students and maybe this fact could be the reason for a higher prevalence of use [8,9] with respect to other disadvantaged groups. Both the ECP access price (around EUR 20) and knowledge as to how to access it may condition its use [8,24]. More than half of the participants considered that ECPs should be free, with no significant difference in opinion between the students’ sex. The most common type of ECP used was levonorgestrel, as also indicated in other studies conducted in other countries [30,31,32], despite it being proved that UPA is more effective in preventing pregnancy [33]. Our results showed that participants were not aware of the existence of an ECP that is effective up to 5 days after the risk of pregnancy sex, with the mechanism of action of an ECP.

It was observed that those students having sexual intercourse at an earlier age had used EC in a larger proportion than those who had delayed their first sexual experience. This could be due to higher sexual risk behavior at earlier ages [19,21,34]. The onset of sexual intercourse of the sample is consistent with the average Spanish youth [13].

In line with results obtained by Jiménez-Iglesias, users of the ECP in this study did not change their usual contraceptive method after ECP use [12]. As other studies with young people have proved, condoms are the most commonly used contraceptive method, and the main cause of EC use is the condom breaking/slipping or not using any contraception method at all [7,21,30,34,35], showing similar results to this study with university students. In the light of the results obtained, and the high percentage of users who declared to have experienced a condom break/slip, it is necessary to reinforce education on the correct use of this contraceptive method in order to avoid this recurrence. Moreover, it is necessary to place particular emphasis on the fact that coitus interruptus is not a contraceptive method to prevent unintended pregnancy.

Studies conducted in the United Kingdom and the USA on the contraceptive consumption profile showed that it was more used among single women [9,17,36] than among those who had a partner, those who had had an abortion in the last 5 years [8], and those women who used less consistent contraceptives [8,24]. However, this study shows how most participants had a partner when they used EC and were using a condom as a contraceptive method during sexual intercourse [4]. Only three participants had undergone a voluntary abortion procedure, and all of them had used the ECP at least once and had early sexual relationships compared to the average.

As shown in previous studies, the use of EC took place within the first 24 h after unprotected sexual intercourse [4], and mainly within the first 12 h, with no differences observed regarding the student’s age [30]. Three out of ten women declared to have taken the ECP more than once, similar to results obtained in other European countries [31,37], and this shows the need for reinforcing the information provided about the correct use of contraceptive methods and thus avoiding repeated use of EC.

It has been observed that awareness, attitudes, and beliefs influence contraceptive use [38]. As shown in similar researches, though awareness of EC is ample, the students’ knowledge on EC can be improved [20,22,27,39]. In particular, the extended beliefs on its efficacy, side effects, and attitudes towards ECP, condition its use [8,10]. In our study, we observed the erroneous beliefs regarding the ECP’s mechanism of action, a question already identified in other studies with nursing students [40]. The belief that EC intake affects women’s fertility in the future has been revealed in previous studies [17,22], and it is in line with our results, as only half of the participants knew that the use of the ECP does not necessarily affect women’s fertility or have consequences in the long term due to repeated use [2,41,42]. There is no evidence of efficacy loss due to repeated use, but this erroneous belief seems widely spread among participants [17]. Though some studies point to the fact that young women are better informed about EC [7], this study cannot confirm this evidence, in light of the results obtained [43].

There is a correlation between having received previous information on ECP and its use [8], a result in accordance with the findings of this study. Moreover, this information was more effective among young women, who showed better knowledge [39], but our study found no difference between male and female students regarding their knowledge on EC, both groups obtaining very similar scoring. The major sources of information were health professionals and the Internet, while other studies indicated the school [44], Internet [45], or friends [22] were major information sources.

In this study, male students were asked about their knowledge, attitudes, and experiences regarding EC, taking as a reference their sexual partner as the one who took the ECP. The study conducted by Richards et al. (2016) already revealed that young men wanted to be involved in the decision making regarding contraception in their relationships, and also showed interest in knowing more about EC [46]. This fact is confirmed in this study, where they actively participated with their answers and showed a similar knowledge level to that of their female peers in almost all items. It is therefore necessary to foster and encourage their participation in all formative initiatives aimed at the youth.

### Limitations

This study was conducted using a sample of university students in southern Spain, and, thus, similar results cannot be expected for other knowledge areas or other regions in the country. As students related to health sciences, it is possible to conclude that they may be more knowledgeable and possess a healthier attitude towards this subject [13,47] than those students focusing in other studies. Furthermore, the absence of cultural diversity in the sample does not allow exploration of this aspect. Due to the cross-sectional nature of the study, causality cannot be proved.

## 5. Conclusions

All university students showed an awareness about EC and, in general, a positive attitude towards its use. One out of three university students who used the ECP, showed a lack of knowledge regarding its mechanisms of action, efficacy, and the types of ECP. This study helps to define the formative contents regarding the ECP aimed at university students in order to ensure their sex education and information on emergency contraception is adequate. It is necessary to address the issues detected both as users and health professionals who will advise others in the future, so that the information they provide is based on scientific evidence.

## Figures and Tables

**Table 1:** Socio-demographic characteristics.

Socio-Demographic Variables	Total % (*n*) 100 (478)	Men ^1^ % (*n*) 18.8 (90)	Women % (*n*) 81.2 (288)	*p*-Value
Age (years), Mean (SD)	20.87 (4.52)	22.13 (6.48)	20.58 (3.88)	0.003
Sexual orientation, % (*n*)				
Heterosexual	86.2 (412)	84.3 (75)	87.1 (337)	
Homosexual	3.1 (15)	10.1 (9)	1.6 (6)	n.a. ^2^
Bisexual	10.3 (49)	5.6 (5)	11.4 (44)	
Have you had any intercourse? Yes, % (*n*)	83.9 (401)	93.3 (84)	81.7 (317)	0.006
Age of first sexual intercourse, Median (SD)	16.53 (1.46)	16.99 (1.57)	16.41 (1.41)	0.001
Received any information on the ECP, Yes, % (*n*)	70.5 (337)	74.4 (67)	69.6 (270)	0.442

^1^ Male students answered question in relation to their sexual partners. ^2^ Not applicable.

**Table 2:** Knowledge about ECP by sex.

Item	Men	Women	*p*-Value
	Correct (*n*)%	Correct (*n*)%	
1. ECP are abortive, lead to embryo abortion, False	58.9 (53)	75.5 (293)	0.001
2. A medical prescription is needed to purchase the postcoital pill, False	82.2 (74)	87.9 (341)	0.167
3. If the woman is pregnant, the pill may harm the zygote or embryo, False	48.9 (44)	46.6 (181)	0.726
4. The ECP protect against sexual transmission infections (STI), False	100 (90)	98.2 (381)	0.357
5. There is one type of oral emergency contraception that can be used within 5 days after risk of pregnancy sex, True	38.9 (35)	35.3 (137)	0.543
6. Morning-after pill affects women’s reproductive health in the long term, reducing the possibilities to conceive on later life stages, False	50 (45)	49.7 (193)	1.000
7. Efficacy of postcoital contraception is reduced each hour after risk of pregnancy sex, True	75.6 (68)	78.4 (304)	0.575
8. A pregnancy test is required prior to take ECP, False	83.3 (75)	86.1 (334)	0.507
9. Postcoital pills can be used whenever necessary, without affecting its efficacy, True	5.6 (5)	6.7 (26)	0.816
10. ECP is a contraceptive method, False	50 (45)	66.8 (259)	0.004

**Table 3:** Risk factors associated to ECP use.

	Crude OR (95% CI)	*p*-Value	Adjusted OR (95% CI)	*p*-Value
Age * (older than 20 years)	3.08 (1.98; 4.79)	<0.001	3.04 (1.92; 4.79)	<0.001
Sex (Woman)	2.90 (1.53; 5.47)	0.001	2.87 (1.47; 5.61)	0.002
Had you ever received any information/guidance on postcoital contraception? (Yes)	1.26 (0.77; 2.06)	0.35		
Contraceptive Methods ** (Withdrawal/No method)	2.02 (1.31; 3.12)	0.001	1.90 (1.20; 3.01)	0.006
Total score	1.19 (1.05; 1.37)	0.008	1.16 (1.01; 1.34)	0.032

* Age variable was stacked in younger and older than 20 years old. ** Contraceptive methods variable was stacked in users of consistent contraceptive use and users of withdrawal/no method at all.

## Data Availability

All data generated or analyzed during this study are included in this published article.
